# The Instrumentation of a Microfluidic Analyzer Enabling the Characterization of the Specific Membrane Capacitance, Cytoplasm Conductivity, and Instantaneous Young’s Modulus of Single Cells

**DOI:** 10.3390/ijms18061158

**Published:** 2017-06-19

**Authors:** Ke Wang, Yang Zhao, Deyong Chen, Chengjun Huang, Beiyuan Fan, Rong Long, Chia-Hsun Hsieh, Junbo Wang, Min-Hsien Wu, Jian Chen

**Affiliations:** 1 State Key Laboratory of Transducer Technology, Institute of Electronics, Chinese Academy of Sciences, Beijing 100190, China; wangke2014@mails.ucas.ac.cn (K.W.); dychen@mail.ie.ac.cn (D.C.); fanbeiyuan@ucas.ac.cn (B.F.); 2 School of Electronic, Electrical and Communication Engineering, University of Chinese Academy of Sciences, Beijing 100190, China; 3 Institute of Microelectronics of Chinese Academy of Sciences, Beijing 100029, China; zhaoyang@ime.ac.cn (Y.Z.); huangchengjun@ime.ac.cn (C.H.); 4 Department of Mechanical Engineering, University of Colorado, Boulder, CO 80309, USA; Rong.Long@colorado.edu; 5 Division of Haematology/Oncology, Department of Internal Medicine, Chang Gung Memorial Hospital at Linkou, Taoyuan City 33302, Taiwan; wisdom5000@gmail.com; 6 Graduate Institute of Biochemical and Biomedical Engineering, Chang Gung University, Taoyuan City 33302, Taiwan

**Keywords:** instrumentation, microfluidics, single-cell analysis, specific membrane capacitance, cytoplasm conductivity, instantaneous Young’s modulus

## Abstract

This paper presents the instrumentation of a microfluidic analyzer enabling the characterization of single-cell biophysical properties, which includes seven key components: a microfluidic module, a pressure module, an imaging module, an impedance module, two LabVIEW platforms for instrument operation and raw data processing, respectively, and a Python code for data translation. Under the control of the LabVIEW platform for instrument operation, the pressure module flushes single cells into the microfluidic module with raw biophysical parameters sampled by the imaging and impedance modules and processed by the LabVIEW platform for raw data processing, which were further translated into intrinsic cellular biophysical parameters using the code developed in Python. Based on this system, specific membrane capacitance, cytoplasm conductivity, and instantaneous Young’s modulus of three cell types were quantified as 2.76 ± 0.57 μF/cm^2^, 1.00 ± 0.14 S/m, and 3.79 ± 1.11 kPa for A549 cells (*n*_cell_ = 202); 1.88 ± 0.31 μF/cm^2^, 1.05 ± 0.16 S/m, and 3.74 ± 0.75 kPa for 95D cells (*n*_cell_ = 257); 2.11 ± 0.38 μF/cm^2^, 0.87 ± 0.11 S/m, and 5.39 ± 0.89 kPa for H460 cells (*n*_cell_ = 246). As a semi-automatic instrument with a throughput of roughly 1 cell per second, this prototype instrument can be potentially used for the characterization of cellular biophysical properties.

## 1. Introduction

Biophysical properties of single cells are mainly determined by cytoskeletons and cellular membranes [1,2]. Variations in cellular biophysical properties are closely related to various physiological and pathological processes of blood cells, tumor cells, and stem cells [3,4,5].

Well-established approaches for measuring electrical properties (e.g., specific membrane capacitance (C_specific membrane_) and cytoplasm conductivity (σ_conductivity_)) of single cells include electrorotation, patch clamping, impedance spectroscopy, and dielectrophoresis [6]. In electrorotation, rotating electric fields are exerted to rotate suspended single cells, and the corresponding cellular rotating parameters are translated to intrinsic electrical properties [7]. From the perspective of technical developments, automation of electrorotation was demonstrated where cellular rotational motions were processed automatically [8,9]. In addition, large-array electrodes were fabricated to conduct electrorotation of single cells in parallel [10,11]. Although tremendous work was conducted, electrorotation still suffers from limited low throughput since it is incapable of measuring single cells continuously (e.g., flow cytometry). 

In patch clamping, moreover, a voltage signal is applied to the patched cellular membrane portion and the corresponding impedance data are interpreted to cellular membrane capacitances [12]. From the perspective of instrument and measurement, further developments focused on the improvements in measurement accuracies [13,14] and automation [15,16]. Although patch-clamping techniques enable time-resolved measurements of cellular membrane capacitances, it is a low-throughput approach due to the tedious process of patch formation. Therefore, such a technique cannot collect membrane capacitance from a large number of cells.

In impedance spectroscopy, single cells are confined between two electrodes where a frequency-dependent excitation signal is applied to measure the corresponding current responses. Although this approach can collect multiple-frequency impedance data of single cells, these raw data cannot be effectively translated to C_specific membrane_ and σ_conductivity_ due to difficulties in electrical modeling [17,18]. In dielectrophoresis, electrical signals with a group of frequencies are applied around two electrodes and the numbers of attached cells on electrodes at individual frequencies are counted and translated to C_specific membrane_ and σ_conductivity_ based on corresponding cellular dielectrophoretic models. However, this approach cannot characterize electrical properties of cells individually [19,20].

On the other hand, micropipette aspiration and atomic force microscopy (AFM) are well established tools for measuring single-cell mechanical properties (e.g., instantaneous Young’s modulus (E_instantaneous_) and Equilibrium Young’s Modulus (E_equilibrium_)) [6]. In micropipette aspiration, a cell is deformed by applying suction through a micropipette placed on the surface of the cell to infer the cellular elastic responses, based on recorded geometrical changes [21]. Equivalent mechanical models of single cells were further proposed for data translation in order to increase the measurement accuracy [22,23]. In addition, automatic setup was recently developed to increase the processing throughput [24,25]. However, the throughput of micropipette aspiration is still limited due to the complicated process of cell manipulation, rending the collection of mechanical properties of a large number of cells impossible.

In AFM, a probe tip is pressed into the cellular surface and the corresponding probe deflection is translated to cellular stiffness [26]. Further technical developments in AFM addressed the issue of measurement accuracies by evaluating the side effects of key probe tip parameters (e.g., tip indentation speed, depth, and frequency) on measurement results [27,28]). Although AFM functions as a scientific instrument and enables the characterization of cellular mechanical properties in its physiological state [29], it normally suffers from the low throughput problem since multiple scans on the same cell are required to produce accurate measurement results of cellular mechanical properties.

In summary, the conventional approaches for measuring cellular biophysical properties (e.g., patch clamping and micropipette aspiration) generally suffer from limited assay throughput. In addition, they can only collect either electrical or mechanical properties of single cells [30]. In order to address these issues, we aspirated single cells to the entrance of a microfabricated pipette with impedance recorded as a combination of patch clamping and micropipette aspiration [31]. This measurement strategy can collect two types of biophysical parameters of single cells, which, however, can only report results from tens of single cells due to limitations in working throughput. 

Furthermore, we demonstrated the concept of using a higher pressure to aspirate individual cells through microfabricated pipettes continuously with cellular traveling impedance monitored to measure cellular electrical/mechanical properties continuously [32,33]. Based on the developed equivalent electrical/mechanical models, the specific membrane capacitance and instantaneous Young’s modulus of hundreds of single cells were obtained [34]. 

In this study, the instrumentation of the aforementioned concept was demonstrated where both instrument operation and data processing were conducted in a semi-automatic manner. In comparison with the conventional techniques (e.g., patch clamping and micropipette aspiration), the equipment developed in this study aspirated and characterized single cells in a continuous manner, which can produce a throughput of two orders higher (e.g., roughly 1 cell per second). In Section 2, working mechanisms and key units of the developed instrument are described. In Section 3, the functionalities of individual modules are described in detail. In Section 4, biophysical property characterization for three types of tumor cells using the developed instrument is demonstrated. Conclusion and future developments are included in Section 5.

## 2. Schematics and Working Mechanism

**Figure 1:**
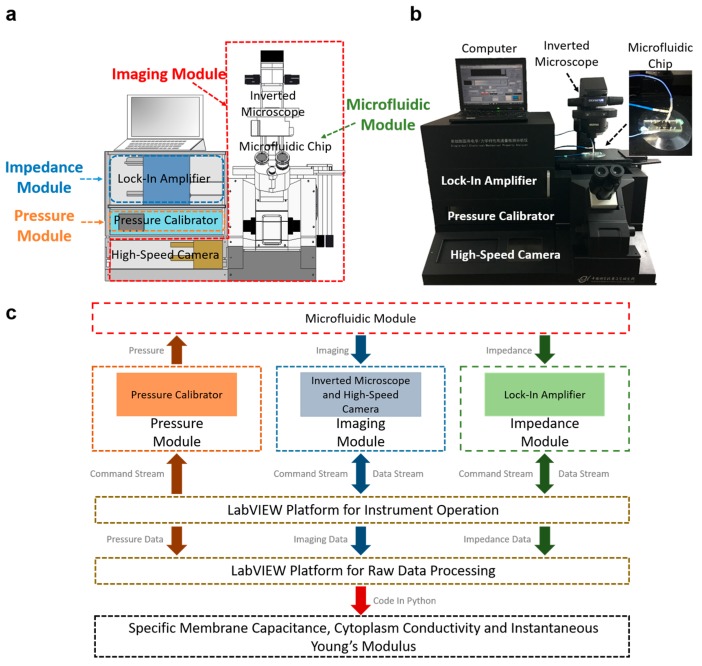
(**a**) Schematics and (**b**) prototype of the microfluidic system enabling biophysical property characterization of single cells. The developed instrument consists of seven key units, including a microfluidic module composed of a constriction channel-based microfluidic device, a pressure module composed of a pressure controller, an imaging module composed of an inverted microscope and a high-speed camera, an impedance module composed of an impedance analyzer, two LabVIEW platforms for instrument operation and raw data processing, and a code in Python for data translation, respectively. (**c**) Working flow chart of the developed microfluidic instrument. Under the control of the LabVIEW platform for instrument operation, the pressure module flushes cells in suspension into the constriction channel of the microfluidic module, while the cellular entry and traveling processes are simultaneously monitored by the imaging and the impedance modules. In data processing, raw biophysical data including elongation length during the process in which a cell enters into the constriction channel and impedance values during the process in which a cell travels into the constriction channel were obtained by the LabVIEW platform for raw data processing, which were further translated to intrinsic biophysical parameters of C_specific membrane_, σ_conductivity_, and E_instantaneous_, leveraging the code in Python.

Figure 1a,b schematically illustrate the prototype of the developed microfluidic instrument enabling biophysical property characterization of single cells. The instrument consists of seven key units, including a microfluidic module composed of a constriction channel, a pressure module composed of a pressure controller, an imaging module composed of an inverted microscope and a high-speed camera, an impedance module composed of an impedance analyzer, two LabVIEW platforms for instrument operation and raw data processing, and a code in Python for the acquisition of intrinsic biophysical parameters of C_specific membrane_, σ_conductivity_, and E_instantaneous_.

The flow chart of the developed microfluidic instrument is shown in Figure 1c. Under the control of the LabVIEW platform for instrument operation, the pressure module flushes cells in suspension into the constriction channel of the microfluidic module with cellular entry and traveling processes monitored by the imaging and the impedance modules. In data processing, raw biophysical data including the elongation of cells when they enter the constriction channel and the impedance values when individual cells travel in the constriction channel were obtained by the LabVIEW platform for raw data processing. These data were further translated to intrinsic biophysical parameters C_specific membrane_, σ_conductivity_, and E_instantaneous_, based on the code in Python.

## 3. Functionalities of Individual Modules

### 3.1. Microfluidic Module

**Figure 2:**
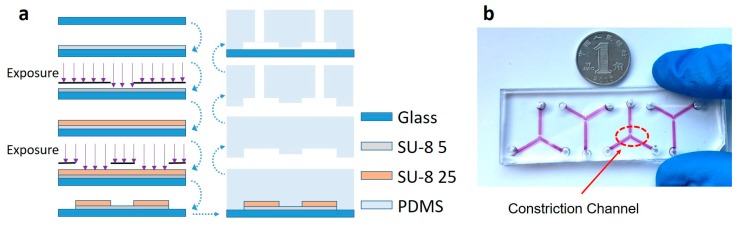
Fabrication process (**a**) and a prototype device (**b**) of the microfluidic module. In the microfluidic module, a micro device with a constriction channel was fabricated using conventional soft lithography including key steps of SU-8 exposure, PDMS molding and bonding between patterned PDMS with a glass slide.

The microfluidic module consists of a constriction channel (cross-section area: 10 μm × 10 μm) in a polydimethylsiloxane (PDMS) elastomer (Dow Corning Corp., Aubur, MI, USA), which was replicated from a double-layer SU-8 mold master (MicroChem Corp., Westborough, MA, USA) (see Figure 2a). Briefly, SU-8 5 was spin-coated and exposed without development to form the constriction channel with a height of 10 μm. Then, SU-8 25 (cell loading channel with a height of 25 μm) was spin-coated on top of the first SU-8 layer, exposed with alignment and developed, forming the two-layer mold master. PDMS prepolymers and curing agents (10:1 in weight) were mixed, poured on channel masters, and baked in an oven for crosslinking. PDMS channels were then peeled from the SU-8 masters, punched to form reservoir holes, and bonded to glass slides after plasma treatment. The fabricated microfluidic devices were shown in Figure 2b, where multiple constriction channels can be fabricated in one device.

### 3.2. Instrument Operation

Figure S1 shows the LabVIEW platform for instrument operation, which regulates the pressure, imaging, and impedance modules. For the regulation of the impedance module (7270, Signal Recovery, Berwyn, PA, USA), a two-layer interface was adopted; in the first layer (Figure S1a), key parameters including impedance frequencies and sampling rates were defined, while in the second layer (Figure S1b), two-frequency impedance values were collected and properly stored. For detailed operation conditions, please refer to [34].

In regulating the pressure module, the pressure value generated by the pressure calibrator (DPI-610, Druck, Boston, MA, USA) was defined in the LabVIEW platform with the real-time pressure values measured and displayed (see Figure S1b). In controlling the imaging module, key parameters including exposure times, image resolutions, and sampling rates were effectively regulated by the LabVIEW platform where real-time images of the cell entry and traveling processes were displayed and stored (see Figure S1b).

Based on this LabVIEW platform, the operations of the pressure, imaging, and impedance modules were well coordinated to simultaneously collect both impedance and imaging data of cells under measurement (see Supplementary Video I). Figure S1c–f show the experimental results with images recorded at 200 fps and impedances recorded at 25 points per second under a pressure of 1 kPa. More specifically, Figure S1c–e record an incoming cell with cellular entry and traveling processes monitored and Figure S1f displays the collected impedance values at the end of one experimental cycle where each dip represented one cell under measurement.

### 3.3. Data Processing

Figure S2a shows the LabVIEW platform for the collection of raw cellular biophysical data, including the elongation length of a cell during its entry into the constriction channel (right) and the two-frequency impedance values when the cell was traveling in the constriction channel (left). More specifically, for impedance data, the time period for the traveling of a single cell in the constriction channel was first identified based on the increase in amplitude and then the maximal amplitude values were found and recorded. As for imaging processing, key steps of frame differencing, thresholding, and edge detection were integrated within this platform. For detailed information of data processing, please refer to [34].

This LabVIEW platform enabled the simultaneous processing of both imaging and impedance data (see Supplementary Video II). As an example, Figure S2b shows the acquisition of impedance values for a cell under measurement, and Figure S2c–e show the processed cellular elongation length as the same cell entered into and traveled within the constriction channel.

The translations of raw biophysical parameters into intrinsic cellular biophysical parameters of C_specific membrane_, σ_conductivity_, and E_instantaneous_ were realized by a Python code. The quantification of C_specific membrane_ and σ_conductivity_ was achieved by solving two equations derived from a previously developed equivalent electrical model [34] for the cellular traveling process within the constriction channel. Meanwhile, the quantification of E_instantaneous_ was enabled by solving two equations derived from a previously developed equivalent mechanical model [34] for the cellular entry process into the constriction channel. 

## 4. Demonstration

**Figure 3:**
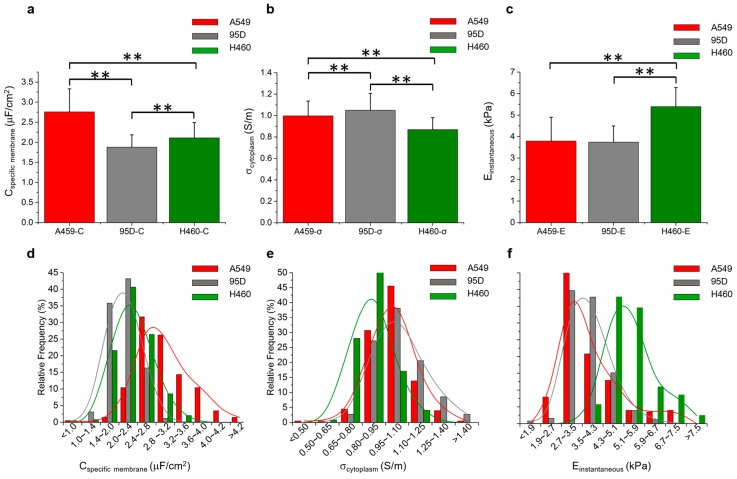
Quantified C_specific membrane_ (**a**), σ_conductivity_ (**b**), and E_instantaneous_ (**c**) for A549 (*n*_cell_ = 202), 95D (*n*_cell_ = 257) and H460 cells (*n*_cell_ = 246) with statistically significant differences represented by ** (*p* < 0.01, multiple group comparisons based on ANOVA). The percentage distributions of C_specific membrane_ (**d**), σ_conductivity_ (**e**), and E_instantaneous_ (**f**) of A549, 95D, and H460 cells further confirm the existence of biophysical property differences of these cell types.

As a functional demonstration of the developed microfluidic instrument, the biophysical properties of three types of lung tumor cells were measured (see Figure 3) where C_specific membrane_, σ_conductivity_, and E_instantaneous_ were quantified as 2.76 ± 0.57 μF/cm^2^, 1.00 ± 0.14 S/m, and 3.79 ± 1.11 kPa for A549 cells (*n*_cell_ = 202); 1.88 ± 0.31 μF/cm^2^, 1.05 ± 0.16 S/m, and 3.74 ± 0.75 kPa for 95D cells (*n*_cell_ = 257); 2.11 ± 0.38 μF/cm^2^, 0.87 ± 0.11 S/m, and 5.39 ± 0.89 kPa for H460 cells (*n*_cell_ = 246). The results were consistent with previous publications [35,36] and the throughput was estimated as 1 cell per second, and thus it is capable of measuring the biophysical properties for hundreds of single cells. Both statistically significant differences (multiple group comparisons based on ANOVA) and percentage distribution differences of C_specific membrane_, σ_conductivity_, and E_instantaneous_ were identified among three lung tumor cell lines, which indicates the possibility of classifying cell types based on their biophysical properties. In addition, a neural network was used for cell classification [32], producing success rates of 87.0% (A549 vs. 95D), 86.6% (A549 vs. H460), 88.0% (95D vs. H460), and 80.2% (A549 vs. 95D vs. H460) (see Figure S3) when these three biophysical parameters were used. These results further confirmed the possibility of classifying cell types based on cellular biophysical properties.

## 5. Conclusions and Future Work

In this study, the instrumentation of a microfluidic analyzer was demonstrated, which enabled the characterization of single-cell biophysical properties at a throughput of roughly 1 cell per second. The functionalities of individual modules were validated and integrated to form the prototype instrument, where the biophysical properties of three types of tumor cells were collected and reported. Future developments of the instrument are aimed at the extraction of more biophysical parameters (e.g., specific membrane capacitance, membrane conductivity, cytoplasm conductivity, instantaneous Young’s modulus, equilibrium Young’s modulus) at a higher throughput (i.e., 1000 cells per second).

## Supplementary Materials

Supplementary materials can be found at www.mdpi.com/1422-0067/18/6/1158/s1.
