# Supplementary material for: The Instrumentation of a Microfluidic Analyzer Enabling the Characterization of the Specific Membrane Capacitance, Cytoplasm Conductivity, and Instantaneous Young’s Modulus of Single Cells
Source: Int J Mol Sci. 2017 Jun 19;18(6):1158. doi: 10.3390/ijms18061158 (PMC5485982; doi:10.3390/ijms18061158)
Supplement: Supplementary file 1 [file ijms-18-01158-s001.zip › ijms-193900-supp/ijms-193900 supplementary .pdf]

International Journal of
Molecular Sciences

The Instrumentation of a Microfluidic Analyzer Enabling the Characterization of the Specific Membrane Capacitance, Cytoplasm Conductivity, and Instantaneous Young's Modulus of Single Cells

Ke Wang, Yang Zhao, Deyong Chen, Chengjun Huang, Beiyuan Fan, Rong Long, Chia-Hsun Hsieh, Junbo Wang, Min-Hsien Wu and Jian Chen

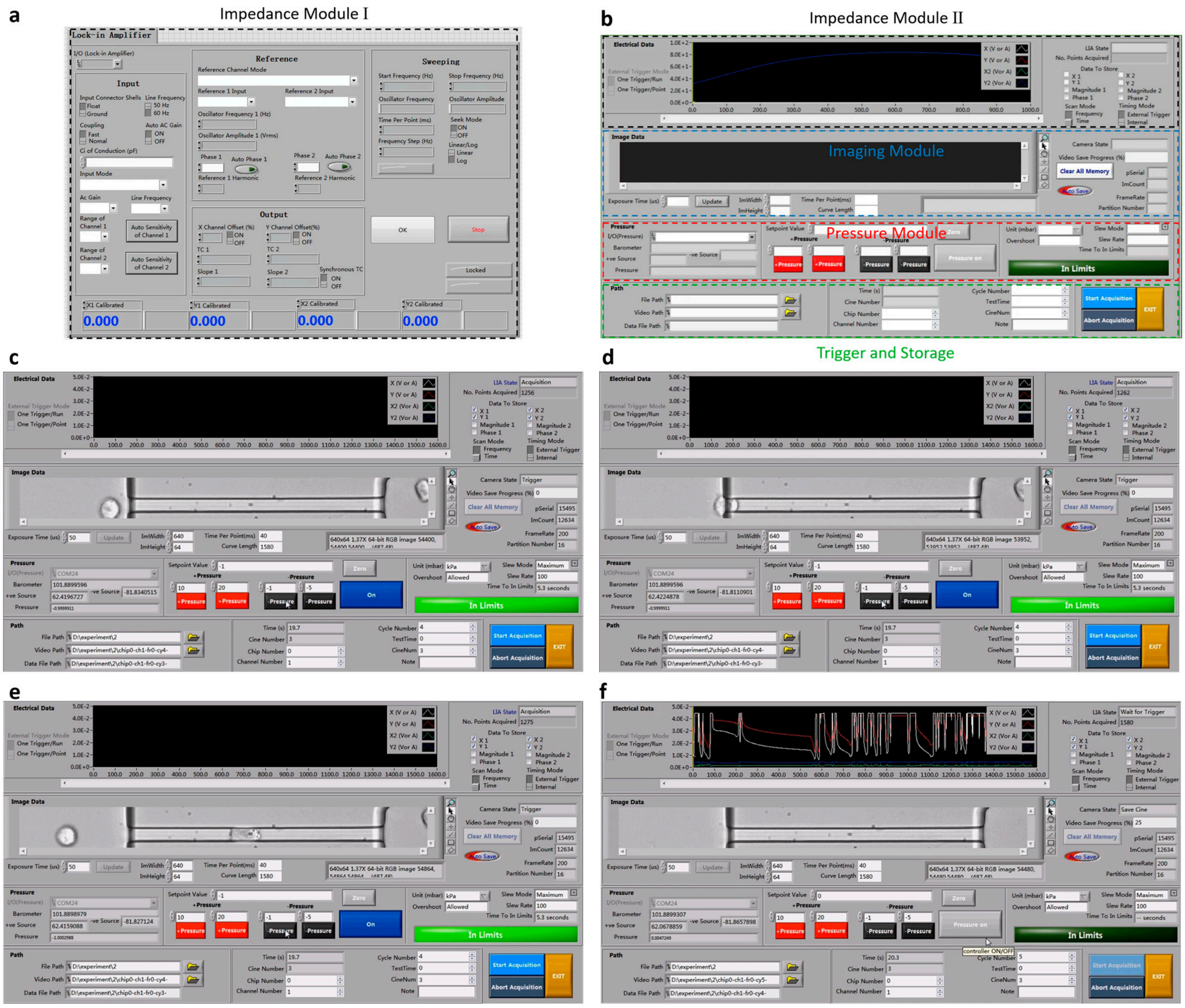

Supplementary Figure 1: (a) and (b) The LabVIEW platform for instrument operation enabling the regulations of the pressure, imaging and impedance modules. (c)-(f) Selected experimental images include a model cell with its entry and travelling process recorded ((c)-(e)) and impedance values collected after one experimental cycle where each dip represented one cell under measurement (f).

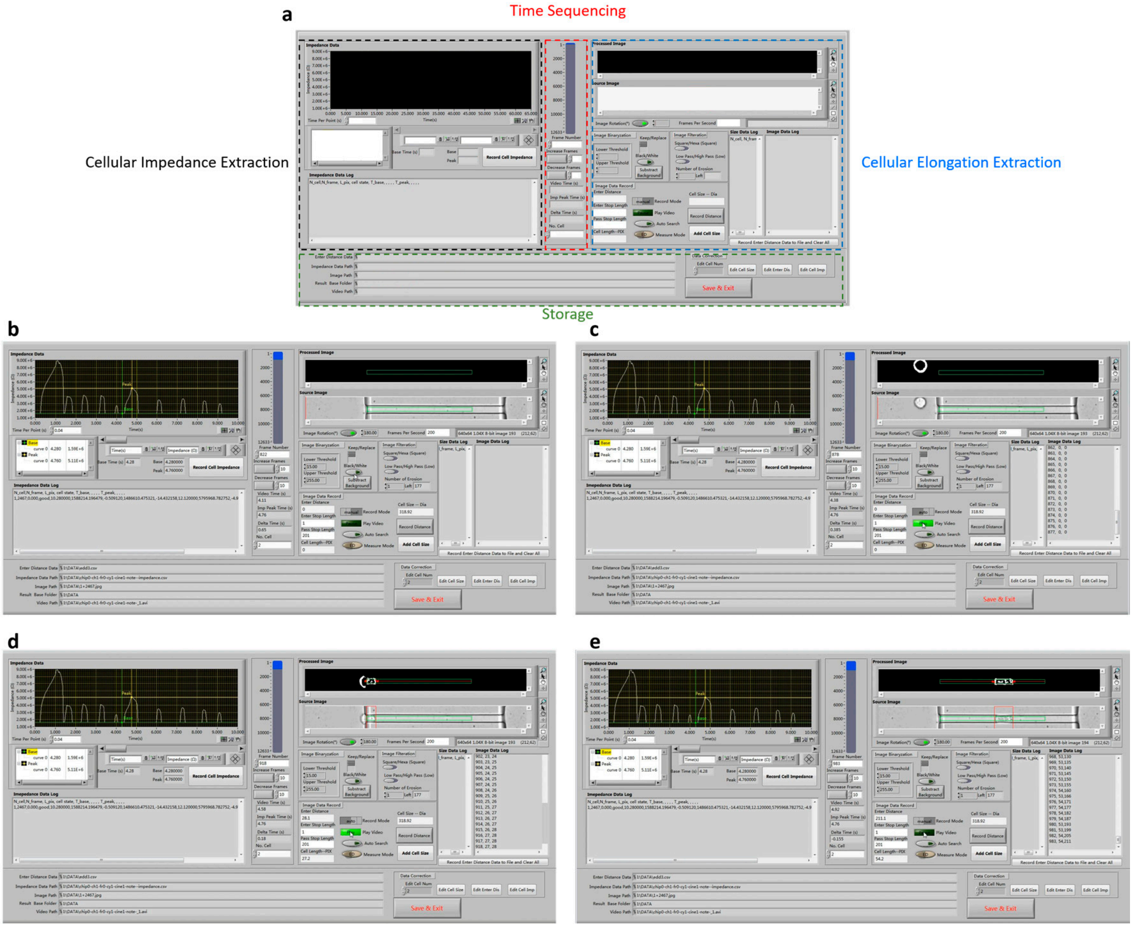

Supplementary Figure 2: (a) The LabVIEW platform for raw data processing, quantifying cellular elongation length during its entry process into the constriction channel (right) and the two-frequency impedance values during the cellular travelling process in the constriction channel (left). (b)-(e) Selected images of a model cell with its impedance values (b), entry elongation length ((c) and (d)) and travelling elongation length (e) quantified.

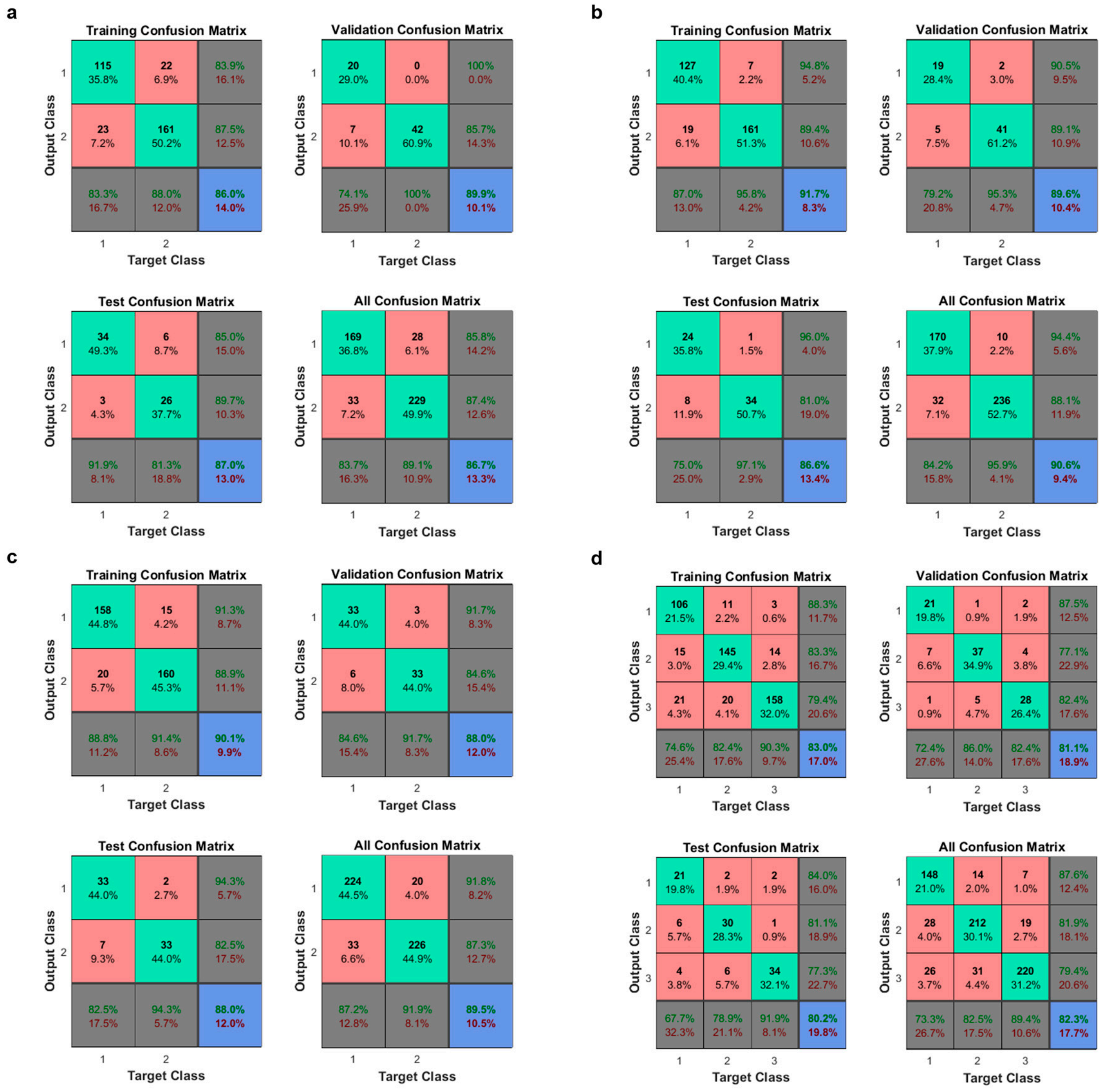

Supplementary Figure 3: Pattern recognition using neural network for classifying A549 cells (n=202), 95D cells (n=257) and H460 cells (n=246) based on $C_{specific\ membrane}$, $\sigma_{conductivity}$ and $E_{instantaneous}$. (a) Confusion matrix of classifying A549 and 95D cells, producing success rates of 86.0% (training group), 89.9% (validation group), and **87.0%** (test group). (b) Confusion matrix of classifying A549 and H460 cells, producing success rates of 91.7% (training group), 89.6% (validation group), and **86.6%** (test group). (c) Confusion matrix of classifying 95D and H460 cells, producing success rates of 90.1% (training group), 88.0% (validation group), and **88.0%** (test group). (d) Confusion matrix of classifying A549, 95D and H460 cells, producing success rates of 83.0% (training group), 81.1% (validation group), and **80.2%** (test group).
